# ‘Case of the Month’ from Addenbrooke's Hospital, Cambridge, UK: surgery after near complete response to combined immunotherapy and tyrosine kinase inhibitor therapy for metastatic renal cell carcinoma with inferior vena cava tumour thrombus

**DOI:** 10.1111/bju.16472

**Published:** 2024-07-16

**Authors:** James P. Blackmur, James N. Armitage, James O. Jones, Antony C.P. Riddick, Thomas J. Mitchell, William H.J. Ince, Sona Appukutty, Anne Y. Warren, Kate Fife, Brent O'Carrigan, Grant D. Stewart

**Affiliations:** ^1^ Department of Urology Cambridge University Hospitals NHS Foundation Trust Cambridge UK; ^2^ Department of Oncology Cambridge University Hospitals NHS Foundation Trust Cambridge UK; ^3^ Department of Pathology Cambridge University Hospitals NHS Foundation Trust Cambridge UK; ^4^ University of Cambridge Cambridge UK

**Keywords:** Cytoreductive nephrectomy, IVC tumour thrombus, metastatic renal cell carcinoma, immunotherapy, kidney cancer

**Abbreviations**
ccRCC: clear cell RCC
CN: cytoreductive nephrectomy
IMDC: International Metastatic RCC Database Consortium
IO: immunotherapy
ISUP: International Society of Urological Pathology
IVC: inferior vena cava
MDT: multidisciplinary team
PR: pathological response
SACT: systemic anti‐cancer therapy
TKI: tyrosine kinase inhibitor
VTT: venous tumour thrombus

## Introduction

While systemic anti‐cancer therapy (SACT) has become the principal management option for many patients with metastatic RCC, there remains debate on the benefits of delayed cytoreductive nephrectomy (CN), and on whether immediate CN and tumour thrombectomy is the correct strategy when there is venous tumour thrombus (VTT) extending into the inferior vena cava (IVC). Given the poor prognosis associated with untreated VTT, the risks of rapid progression and the sequalae from venous congestion or distal embolism, urgent extirpative surgery may be beneficial, dependent on patient fitness. Alternatively, given the high clinical response rates of modern immunotherapy (IO)‐based systemic treatment, there has been interest in downstaging cases of locally advanced RCC (including those with VTT) with neoadjuvant therapy [1].

We present a case where a patient with metastatic clear cell (cc)RCC and with Mayo Level IV IVC tumour thrombus had an excellent response to treatment with lenvatinib and pembrolizumab, which allowed for single‐cavity extirpative surgery.

## Case Description

**Fig. 1:**
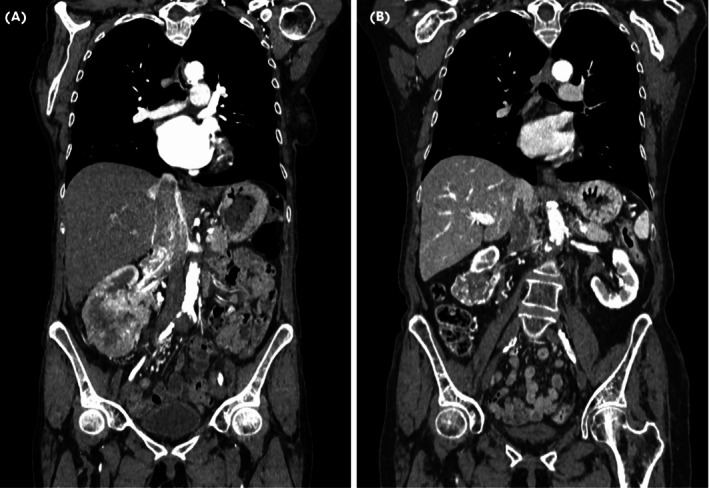
Coronal CT reconstructions at (**A**) baseline showing large right renal mass and supra‐diaphragmatic extension of tumour thrombus in the IVC (Mayo Level IV) and (**B**) at 15 months showing shrinkage of renal mass and tumour thrombus to below level of hepatic veins (Mayo Level II).

A 69‐year‐old female of Eastern European descent presented in October 2022 with lower abdominal pain, constipation, and lethargy. A CT scan (Fig. 1A) identified a 95‐mm right lower pole renal mass, with VTT extending to the right atrium (Mayo Level IV). She also had an enhancing 18‐mm right adrenal nodule and three lung nodules (largest 9 mm) in keeping with metastatic disease. She had a past history of hypertension and smoking (~50 pack‐years) and reported restriction in physical activity due to fatigue/lethargy (Eastern Cooperative Oncology Group [ECOG] Performance Status 1). Initial biochemistry found of note: haemoglobin 84 g/L (reference range: 118–158 g/L), platelets 643 × 10^9^/L (reference range: 160–370 × 10^9^/L), neutrophils 8.23 × 10^9^/L (reference range: 1.50–7.70 × 10^9^/L), corrected calcium 2.65 mmoL/L (reference range: 2.20–2.60 mmoL/L). Overall, she had poor‐risk disease (score 5) per the International Metastatic RCC Database Consortium (IMDC). Ultrasound‐guided renal biopsy was predominantly necrotic with focal International Society of Urological Pathology (ISUP) Grade 4 ccRCC. There was no evidence of sarcomatoid or rhabdoid morphology. After multidisciplinary team (MDT) review, and careful discussion with the patient via translator, as per international guidelines she was treated with lenvatinib and pembrolizumab.

Between 3 and 6 months after treatment initiation, she developed IO‐induced diabetes mellitus (treated with insulin), hypoadrenalism (treated with maintenance hydrocortisone), and Grade 2 diarrhoea, which responded to steroid treatment and pembrolizumab omission.

Repeat CT scan at 6 months showed reduction in the size of the renal mass to 55 mm, along with resolution of the lung nodules and reduction in the extent of the tumour thrombus to the level of the hepatic veins (Mayo Level III). Pembrolizumab was restarted, but between 10 and 14 months after treatment initiation, she developed Grade 3 colitis, Grade 2 hepatitis (peak alanine transaminase 200 U/L [reference range 10–49 U/L]) and had a transient ischaemic attack.

**Fig. 2:**
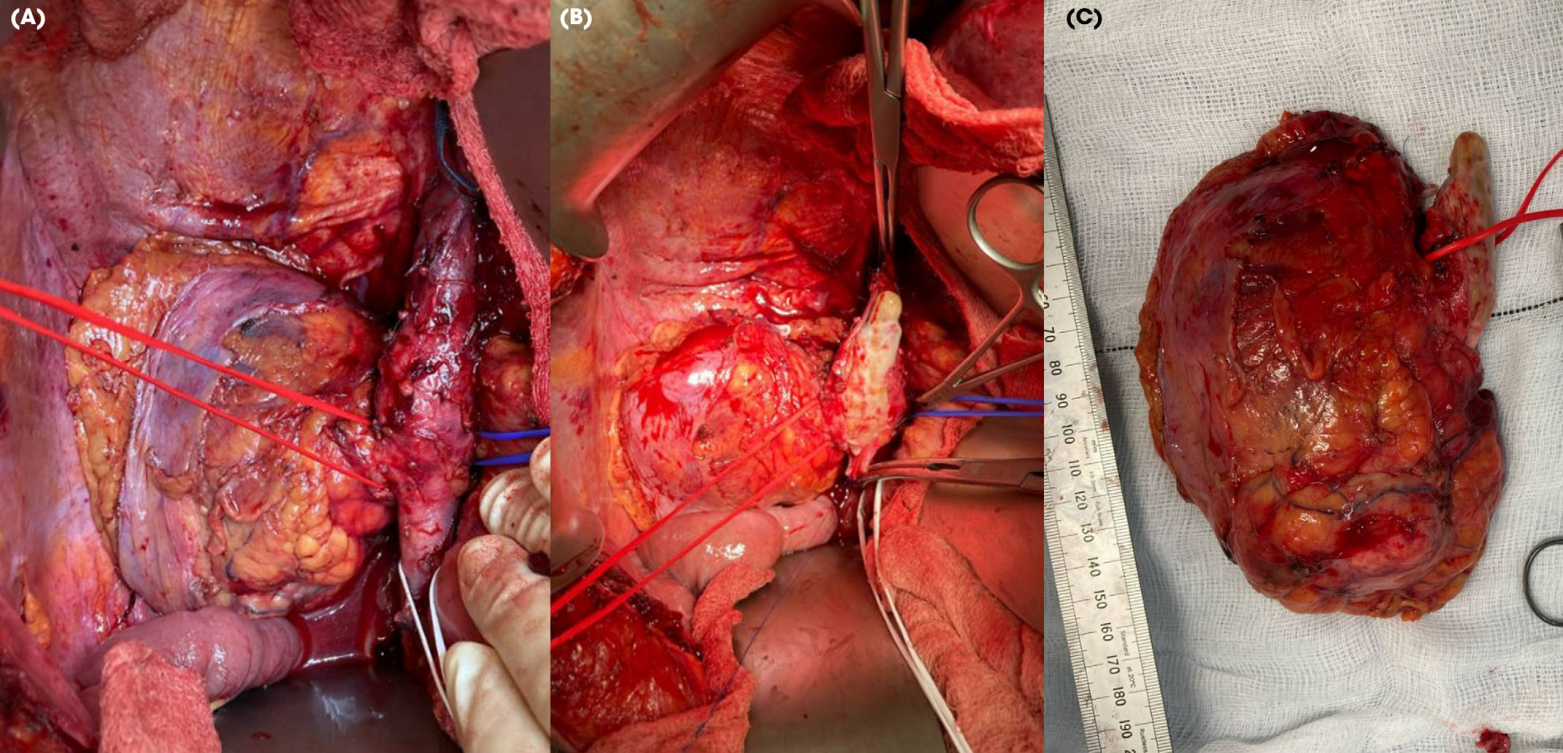
(**A**) Right renal tumour, with sloops on right renal vein (red), left renal vein (blue) and infrarenal IVC (white), (**B**) with clamps applied and with tumour thrombus elevated from cavotomy, and (**C**) right kidney, adrenal gland and tumour thrombus excised in continuity.

A CT scan at 15 months (Fig. 1B) showed minimal further reduction in the size of the renal mass (51 mm), and further shrinkage of the caval tumour thrombus (Mayo Level II), with a new 11‐mm aortocaval node and a stable 15‐mm right adrenal nodule. Given the multiple toxicities on systemic treatment, CN was again discussed with the patient. MRI confirmed the tumour thrombus level extending to 1.2 cm below the right hepatic vein. Following MDT discussion, she proceeded to right open CN, caval thrombectomy, right adrenalectomy and lymphadenectomy 16 months after initiation of SACT. While the case required mobilisation of the liver, caval thrombectomy was successfully undertaken using infra‐renal, left renal and infra‐hepatic caval cross‐clamping (Fig. 2). She made an uneventful recovery and was discharged home on postoperative Day 8.

**Fig. 3:**
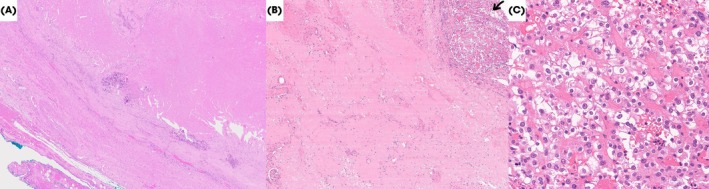
(**A**) Haematoxylin and eosin (H&E; ×5) tumour thrombus showing necrosis, fibrosis and microcalcification. (**B**) H&E (×10) section from kidney showing extensive fibrosis and small foci of residual ccRCC (marked by black arrow) and (**C**) same area showing ccRCC at high power (×40).

Pathology confirmed 95% of the right renal lesion showed fibrosis, haemorrhage, microcalcification and tumour necrosis (Fig. 3). Focally the mass showed ISUP Grade 3 ccRCC with no sarcomatoid or rhabdoid morphology. There were no viable tumour cells within the renal vein or IVC thrombus, and the excised caval wall showed no evidence of malignancy. One lymph node showed ccRCC. The adrenal lesion was described as showing focal scarring and haemosiderin laden macrophages suspicious for prior metastatic focus, with an additional adrenocortical adenoma. Final stage was ypT1a N1. The patient was well at 3 months after surgery, with CT scan showing no evidence of recurrent or metastatic disease.

## Discussion

With the exception of select patients with IMDC favourable‐risk and low‐volume metastatic disease, SACT has become the principal initial treatment option for patients with metastatic RCC [2]. While there are a number of ongoing or upcoming studies investigating the benefits of deferred CN with IO‐based therapy (ClinicalTrials.gov identifiers: NCT03494816, NCT03977571, NCT05753839, NCT05941169, NCT06279403), a subgroup of particular interest are patients with metastatic RCC who have concurrent VTT, particularly involving the IVC. There may be clear benefits from undertaking urgent extirpative surgery in this setting, as is considered optimal for patients with VTT as part of locally advanced disease. Alternatively, in patients with established metastatic disease, the delivery of modern systemic therapy with proven overall survival benefits could be significantly delayed by perioperative morbidity resulting from CN. There is no Level 1 evidence to support the use of neoadjuvant SACT in cases of locally advanced RCC with VTT, or to guide the timing of CN in cases with concurrent VTT, particularly where that involves the IVC.

This case demonstrates that the combination of IO and tyrosine kinase inhibitor (TKI) therapy can effectively downstage patients with VTT and metastatic disease, which facilitated operative management. The patient's case was regularly discussed at MDT meetings; she was initially judged to have poor‐risk, albeit relatively low‐volume, metastatic disease, and not to be fit for bicaval surgery involving cardiopulmonary bypass. Surgery was specifically re‐discussed with the patient at 6 months, and at various points thereafter; however, she opted to continue systemic therapy.

While numerous studies have demonstrated SACT, followed by delayed CN if indicated, is the preferable treatment sequence for many patients with metastatic disease [2], most studies do not comment on how many of their cohort had VTT, or on the extent of that VTT. Neoadjuvant treatment of patients with Level III or IV VTT is particularly attractive as downstaging has the potential to allow less invasive surgical intervention, as described in the case above. Existing data in this space demonstrate what is achievable by TKI alone, with 73% patients across multiple studies having a measurable decrease in VTT length (mean reduction 1.4 cm), 30% having a reduction in VTT level, and possible impacts on survival with little evidence of toxicity [3]. In the NAXIVA trial (ClinicalTrials.gov identifier: NCT03494816), the median (range) change in VTT length at 9 weeks was 20 (34 to 68 mm), with 41% patients having less extensive surgery performed than was planned prior to axitinib [4]. Given that TKI‐IO combined regimens have been shown to have a superior effect on primary tumour and metastases than TKI therapy alone [2], there is compelling rationale for future neoadjuvant trials using these agents. Of note, one case series reported longer operative time and higher blood loss following neoadjuvant treatment [5]. This marries with our experience where many patients who have received IO‐combined therapy prior to CN have an intense inflammatory reaction or fibrotic tissue, particularly around hilar vessels or the IVC. The case described also highlights potential toxicity of TKI‐IO therapy; patients with immune‐related adverse events may be a group to consider CN at a lower threshold, particularly where this is correlated with response to treatment.

We believe the question of whether and when to perform CN in cases with VTT remains an open one [1]. Does downstaging with SACT, particularly with IO‐combined regimens, have a clinically‐relevant impact on staging of the tumour (i.e., less invasive surgery becomes possible)? What sequence of SACT and CN has the greatest effect on survival? What is the optimal time to offer delayed CN? How can we best assess pathological response (PR) following neoadjuvant treatment? Indeed, do patients need nephrectomy if complete PR has been achieved? Conversely, does SACT result in harm caused by the delay to extirpative surgery? Additional challenges are posed by the histological reporting of deferred CN cases as there is no agreed framework or defined criteria for PR (replacement of the tumour with post‐treatment fibrosis, necrosis, and inflammation can lead to discrepancy between macroscopic size and microscopic stage). None of these questions have as yet been adequately answered. While most patients will have objective responses to systemic therapy, some will have primary progression and there is currently no ability to predict who these patients might be [6]. Identifying these patients will require collaboration between interested centres, comparing those who undergo immediate nephrectomy to those who undergo delayed nephrectomy or SACT alone. In this space, trials of deferred CN after IO‐IO or TKI‐IO combinations should include subgroup analyses of patients with VTT.

## Conclusion

We present a case where SACT effectively down staged a patient's VTT and metastatic disease allowing single‐cavity CN to be undertaken. Whether immediate surgery, or initial SACT with delayed CN, is the correct approach for cases of metastatic disease with VTT remains an open question, and there is an urgent need for trials using IO‐combined therapy in this space.

## Disclosure of Interests

Grant D. Stewart has received educational grants from Pfizer, AstraZeneca and Intuitive Surgical; consultancy fees from Pfizer, MSD, EUSA Pharma and CMR Surgical; Travel expenses from MSD and Pfizer; Speaker fees from Pfizer; Clinical lead (urology) National Kidney Cancer Audit and Topic Advisor for the National Institute for Health and Care Excellence (NICE) kidney cancer guideline. Kate Fife has received advisory, consultancy or speaker fees from ESAI, Ipsen, Merck, Eusa, MSD, Regeneron and Sanofi, conference support from Ipsen, MSD and EUSA, and Institutional research funding from Merck, Exelixis. No other conflicts of interest to declare.
